# Tumor microenvironment promotes lymphatic metastasis of cervical cancer: its mechanisms and clinical implications

**DOI:** 10.3389/fonc.2023.1114042

**Published:** 2023-05-10

**Authors:** Yuting Li, Xiaofan Gao, Yibao Huang, Xiaoran Zhu, Yingying Chen, Liru Xue, Qingqing Zhu, Bo Wang, Mingfu Wu

**Affiliations:** ^1^ National Clinical Research Center for Obstetrical and Gynecological Diseases; Department of Gynecology, Tongji Hospital, Tongji Medical College, Huazhong University of Science and Technology, Wuhan, Hubei, China; ^2^ Key Laboratory of Cancer Invasion and Metastasis, Ministry of Education, Wuhan, Hubei, China

**Keywords:** cervical cancer, tumor microenvironment, immune cells, lymphatic metastasis, lymphangiogenesis

## Abstract

Although previous studies have shed light on the etiology of cervical cancer, metastasis of advanced cervical cancer remains the main reason for the poor outcome and high cancer-related mortality rate. Cervical cancer cells closely communicate with immune cells recruited to the tumor microenvironment (TME), such as lymphocytes, tumor-associated macrophages, and myeloid-derived suppressor cells. The crosstalk between tumors and immune cells has been clearly shown to foster metastatic dissemination. Therefore, unraveling the mechanisms of tumor metastasis is crucial to develop more effective therapies. In this review, we interpret several characteristics of the TME that promote the lymphatic metastasis of cervical cancer, such as immune suppression and premetastatic niche formation. Furthermore, we summarize the complex interactions between tumor cells and immune cells within the TME, as well as potential therapeutic strategies to target the TME.

## Introduction

Cervical cancer (CC) ranks fourth among all types of cancers in terms of morbidity and mortality in women, with an estimated 604,000 new cases and 342,000 deaths worldwide in 2020. Furthermore, CC is the most commonly diagnosed cancer in 23 countries and the leading cause of cancer-related death in 36 countries (1). Although most patients with early-stage CC recover well through surgery, patients with recurrent or metastatic CC are rarely treated effectively (2, 3). Therefore, patients with advanced CC have a poor prognosis and low survival rate (4, 5). Lymphatic vessels are one of the crucial routes by which CC cells metastasize, and treatment failure is often associated with lymph node metastasis (6). In the past, lymphatic vessels were considered to play a passive role in metastasis, acting merely as a channel for tumor cells to invade. However, growing evidence has suggested that tumor-associated lymphatic vessels can actively participate in tumor-related pathological processes, thereby promoting the lymphatic metastasis of CC (7). On the one hand, peritumoral lymphatic vessels can boost tumor cell lymphatic invasion; on the other hand, tumor-draining lymphatic vessels promote tumor metastasis by increasing pumping and lymph flow, recruiting tumor cells, providing cancer stem cell niches, and modulating antitumor immune responses (8).

The tumor microenvironment (TME) has been an area of active research in recent years, as it exerts an instrumental influence on tumor progression and metastasis (9). The TME is largely composed of immune cells, fibroblasts, endothelial cells, stromal cells, and extracellular matrix (ECM) (10, 11). It is well-accepted that the interactions between tumors and immune cells are more complex and dynamic than previously thought. Many subtypes of immune cells infiltrating the TME also possess potent tumor-promoting abilities (11, 12). However, the impact of the crosstalk between the TME and immune cells on tumor metastasis remains unclear. A better understanding of the interactions between CC cells and immune cell and their roles in lymphatic metastasis will be beneficial for developing alternative therapies. In this review, we summarize the role of the TME in CC and introduce five main characteristics of the TME that promote lymph node metastasis.

## Characteristics of the TME

**Figure 1:**
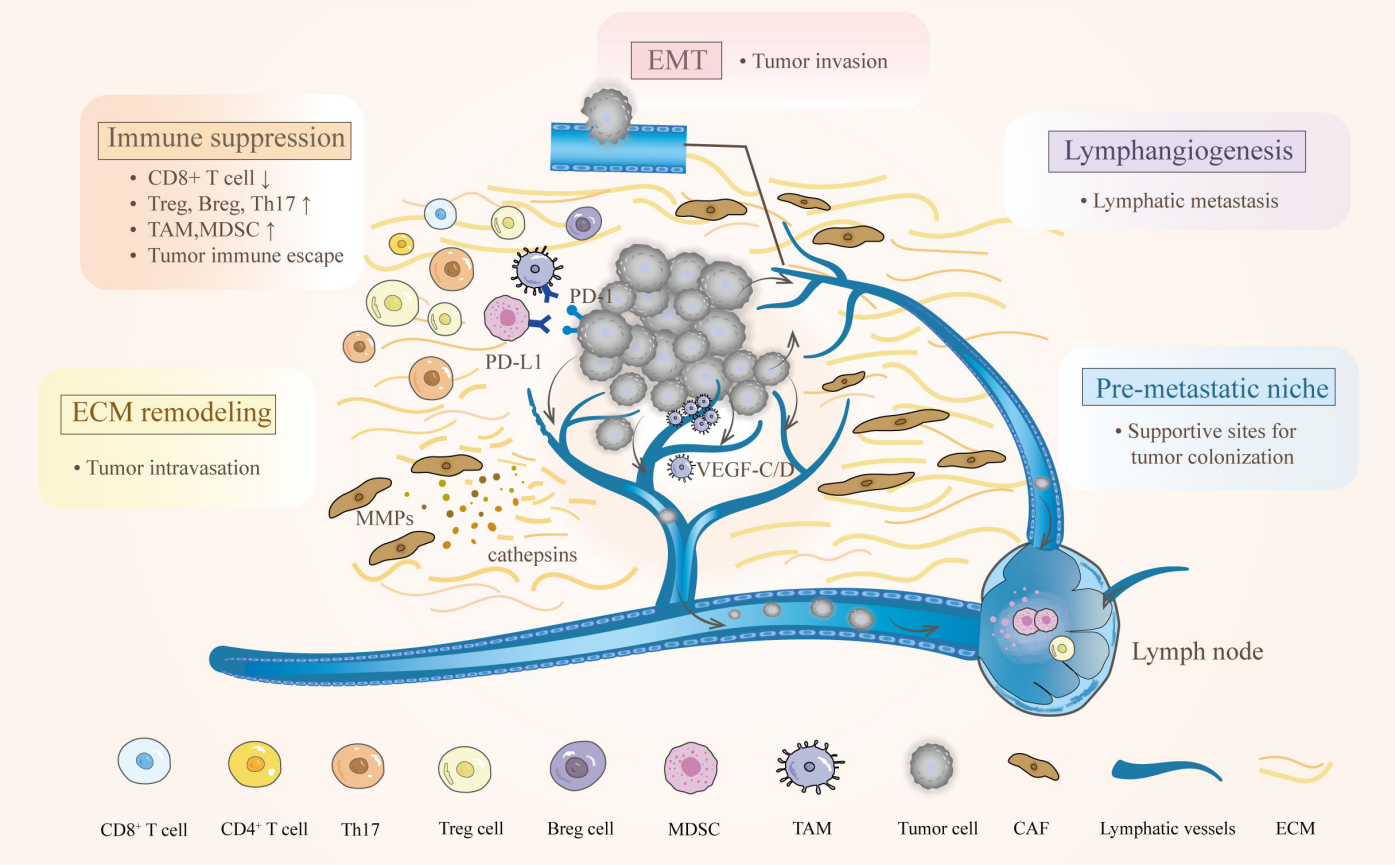
Characteristics of the TME and their effects on lymphatic metastasis in cervical cancer. The complex interactions between cervical cancer and immune cells empower the TME to hamper antitumor immunity. Various bioactive substances derived from primary tumors participate in the regulation of ECM and lymphangiogenesis. After EMT, tumor cells can easily reach and enter the lymphatic vessel. Moreover, creating a premetastatic niche provides supportive sites for metastatic tumor cells.

Successful lymphatic metastasis is a consequence of complex processes involving interactions among multiple components in the TME. The progression can be simplified into four steps: the dissemination of locally invasive tumor cells to the lymphatic vessels, the transport of tumor cells through the lymphatic system to the lymph nodes, the settlement of tumor cells in the lymph nodes, and the growth of micrometastases into a detectable mass (13). Observations of metastasis in afferent lymphatic vessels, as well as the specific and early involvement of the tumor-draining lymph nodes have strongly indicated that tumor cells initiate lymph node metastasis by entering tumor-associated lymphatic vessels (14). Interestingly, the lymphatic system does not act as a passive channel for tumors to invade but actively facilitates lymphatic metastasis in the TME (7, 15). The complicated interplay among tumor cells, immune cells, and the local stroma promotes the lymphatic metastasis of CC. At least five key characteristics of the TME favor this process, including immunosuppression, premetastatic niche formation, lymphangiogenesis, epithelial-mesenchymal transition (EMT), and ECM remodeling (**Figure 1**).

### Immunosuppression

There is a consensus that CC is an immunogenic tumor induced by persistent infection with the high-risk human papillomavirus (HPV) (16). During persistent HPV infection, tumor cells employ a range of strategies for immune evasion. Studies indicate that HPV downregulates the gene expression of C-X-C motif chemokine ligand 14 (CXCL14), the adhesion molecule E-cadherin and Toll-like receptors in host cells, disrupting the recruitment of natural killer (NK) cells, T cells, Langerhans cells and dendritic cells (17, 18). Moreover, HPV impairs the expression of MHC class-I (MHC-I), immunoproteasome subunits, and transporter associated with antigen processing (TAP), interfering with antigen presentation (19). All these strategies enable the virus to escape immunosurveillance and persist for a long time, increasing the risk of lesion progression and malignant transformation.

In addition to the virus, the TME actively participates in tumor proliferation and lymph node metastasis by suppressing antitumor immunity and inducing regulatory and suppressive immune cells. The TME is characterized by an imbalance in T helper (Th) cells in tumor tissues. Normally, CD4^+^ T cells aid cytotoxic CD8^+^ T cells in tumor rejection. However, during the progression of the tumor, the Th1 tumor-suppressive phenotype is converted into a Th2 tumor-promoting phenotype, which hinders the activity of CD8^+^ T cells (12). In fact, reduced intraepithelial infiltration of CD8^+^ T cells and a significantly decreased CD8^+^/CD4^+^ T-cell ratio have been found in CC patients with lymph node metastases (20). Additionally, studies on metastatic tumor-draining lymph nodes in CC have shown that these sites are characterized by suppressor-type cells with significant Th2 polarization and reduced content of effector-type CD8^+^ T cells (21). Recently, researchers have found that the TME exhibits an immunosuppressive state in CC tissue, characterized by infiltration of exhausted CD8^+^ T cells and CD163^+^ CD68^+^ M2-like macrophages (22). Similarly, another study has demonstrated that the cytotoxic activities of CD8^+^ T cells are significantly curtailed by high-level expression of checkpoint genes, such as programmed cell death 1 (PD-1), lymphocyte activating 3 (LAG3) and hepatitis A virus cellular receptor 2 (HAVCR2) (23). All these findings suggest the existence of profoundly immunosuppressive microenvironments in primary tumors and tumor-draining lymph nodes, which protect metastatic tumor cells from immune attacks. Consequently, tumor cells are able to migrate to the lymph nodes.

Notably, specific immune cells, including tumor-associated macrophages (TAMs) and myeloid-derived suppressor cells (MDSCs), contribute to the formation of immunosuppressive TME. Increased infiltration of TAMs is related to a more advanced stage and lymph node metastasis in CC (24–26), as is increased infiltration of MDSCs (27, 28). Both of these cell populations can suppress the proliferation and function of effector T cells and induce the recruitment of regulatory T (Treg) cells and regulatory B (Breg) cells (28–30). Additionally, Th17 cells have been found to promote tumor progression by triggering chronic inflammation within the TME. They are attracted by fibroblasts expressing high levels of C-C motif chemokine ligand (CCL20) (31). Lymphatic endothelial cells (LECs) are also reprogrammed into an essential component with immunosuppressive effects in the TME. In a mouse model, exosome-encapsulated microRNA-1468-5p secreted by CC cells could promote programmed cell death ligand 1 (PD-L1) expression in LECs. Then, the LECs impaired CD8^+^ T-cell immunity by binding to PD-1, enabling CC immune escape (2). Overall, the significant immunosuppressive state offers suitable conditions for tumor proliferation and a window for tumor metastasis.

### Premetastatic niches

Primary tumors can instruct the formation of microenvironments in distant organs hospitable to tumor cells before arriving at these sites. These predetermined microenvironments are termed premetastatic niches (32, 33). Well-established premetastatic niches with a fertile microenvironment support the survival, seeding, colonization, and outgrowth of metastatic tumor cells, resulting in the formation of micrometastases (33). Evidence suggests that the nearest draining lymph nodes in patients with CC can be prepared early to host metastatic cells (15). To successfully build a premetastatic niche, primary CC cells, MDSCs, and other components in the local stromal microenvironment of the host (or future metastatic organ) work together. However, there needs more research to understand the role of CC cells in establishing the premetastatic niche.

The creation of a premetastatic niche is fostered by mobilized and recruited immune cells. These cells remodel the local microenvironment by secreting inflammatory cytokines, growth factors, and proangiogenic molecules (34). For instance, in mouse models, MDSCs have been shown to contribute to premetastatic niche formation by expressing high levels of proinflammatory chemoattractants such as Cxcl2, S100a8, S100a9, and Bv8 (35). Similarly, in ME180 tumor-bearing rats, MDSCs were found to express S100a8/a9 to create a premetastatic niche in the lymph nodes (36). Before metastasis, Treg cells may also condition lymph nodes to form a metastatic niche. Subsequently, PD-L1^+^ M2-like macrophages are recruited to facilitate the expansion of the next wave of Treg cells, preparing for further metastatic spread (37).

Changes in the TME, such as lymphangiogenesis and ECM remodeling, establish a supportive microenvironment that may foster the colonization and outgrowth of metastatic tumor cells in secondary sites (33, 38). Periostin (POSTN) has recently been identified as a crucial matricellular protein in lymph node premetastatic niche development. In a preclinical murine model, cancer-associated fibroblast (CAF)-derived POSTN regulated the function of LECs and promoted the implantation of metastatic cells in the lymph nodes (38, 39). Moreover, both in mouse and human CC, lymphangiogenesis is detected in the tumor-draining lymph nodes even before lymphatic metastasis, confirming the presence of a premetastatic niche (8). The role of lymphatic vessels in promoting metastasis *via* the premetastatic niche has yet to be fully understood. Further analysis of LECs and identification of the key components involved in promoting metastasis *via* lymphangiogenesis may help to unravel these mysteries.

### Tumor lymphangiogenesis

In CC, lymphatic metastasis is one of the major routes of metastasis, within which tumor-associated lymphangiogenesis is an essential event. Tumor lymphangiogenesis involves the migration of endothelial cells into the tumor tissue and the formation of new lymphatic vessels. This process leads to an increase in tumoral lymphatic vessels and enlargement of tumor-draining collecting vessels. The expansion of the lymphatic network promotes tumor migration into the lymphatic circulation and entry into the lymph nodes, where the tumor cells multiply, and potentially facilitate migration to distant organs (14, 40). Clinicopathological studies have shown a positive correlation between lymphatic microvessel density and metastasis (41). As mentioned before, premetastatic lymphangiogenesis in the lymph nodes plays an important role in preparing the site for metastatic dissemination (15). Therefore, lymphangiogenesis and lymphatic remodeling are functionally important in the promotion of tumor metastasis.

Lymphangiogenesis is mediated by various lymphangiogenic factors released by CC cells or host-derived cells within the TME, including vascular endothelial growth factor A (VEGF-A), VEGF-C/D, and angiopoietins. VEGF-C/D promotes tube formation by binding to their receptor VEGFR-3 expressed by LECs (14, 42), while VEGF-A stimulates both angiogenesis and lymphangiogenesis by binding to VEGFR-2 (14). Clinical data indicate elevated VEGF-C expression in peripheral leukocytes and retroperitoneal lymph nodes in CC is associated with tumor progression and lymphatic metastasis (43).

Some tumor-derived factors have been found to promote lymphangiogenesis in CC. For instance, the receptor for activated C-kinase 1 (RACK1), a scaffold protein that participates in many intracellular signal transduction pathways, facilitates tumor invasion and lymphatic tube formation. Upregulated expression of RACK1 promotes lymphangiogenesis *via* galectin-1 (44), which promotes lymphatic vascular growth and contributes to the maintenance of the lymphatic endothelial phenotype (45). In addition, RACK1 interacts with insulin−like growth factor 1 receptor (IGF1R) to promote lymphangiogenesis *via* activation of the AKT/mTOR pathway in CC cells (46). Hematological and neurological expressed 1 (HN1), a microtubule-associated protein, induces lymphangiogenesis by activating the NF-κB signaling pathway (47). Likewise, sine oculis homeobox homolog 1 (SIX1) and protein tyrosine phosphatase receptor type M (PTPRM) expressed in tumor cells both promote tumor lymphangiogenesis by inducing increased expression of VEGF-C (48, 49). Moreover, cancer-secreted exosomal microRNA-221-3p transferred to LECs may promote lymphangiogenesis by downregulating vasohibin-1, an endogenous angiogenesis inhibitor (40). CC-secreted exosomal microRNA-1468-5p also promotes lymphangiogenesis by reprogramming LECs (2). As a regulator of the function of Treg cells, the *Foxp3* gene has a vital role in forming the immunosuppressive microenvironment. Interestingly, a study indicated that Foxp3 was positively correlated with VEGF-C expression and might be involved in lymph node metastasis of CC by promoting lymphangiogenesis (50). These results indicate that multiple tumor-associated factors play important roles in inducing lymphangiogenesis, leading to the lymphatic metastasis of CC.

Other tumor partners in the TME also contribute to lymphangiogenesis in CC. For example, after being cultured in a conditioned medium from CC cell-macrophage coculture, human LECs formed more tube-like structures *in vitro*. These TAMs promoted lymphangiogenesis by increasingly expressing VEGF-C and VEGF-A, which was induced by the interactions with surrounding CC cells (26). Tumor-associated LECs actively participate in lymphatic metastasis by highly expressing soluble semaphorin 4C (sSEMA4C), which promotes lymphangiogenesis by activating PlexinB2-ERBB2 signaling in LECs and facilitates tumor proliferation and migration by activating PlexinB2-MET signaling (7). CAFs regulate LEC functions and promote metastatic cell implantation in the lymph nodes by secreting POSTN, which induces VEGF-C-driven lymphangiogenesis (38). Moreover, cancer-associated inflammation is likely to promote lymphangiogenesis and facilitate metastasis. Studies in CC provide evidence for a clinical association between cyclooxygenase-2 (COX-2) and VEGF-C expression. COX-2, an enzyme required for prostaglandin synthesis, is induced by inflammatory signals in various cell types, including myeloid and endothelial cells. As a consequence, prostaglandins induce the expression of VEGF-C and increase tumor lymphangiogenesis (41, 51). Taken together, these data suggest that various molecules in the TME of CC contribute to tumor lymphangiogenesis. However, it is unclear whether the increased quantity of lymphatic vessels and enlarged draining collecting vessels due to lymphangiogenesis and remodeling are prerequisites for lymphatic metastasis, and the mechanisms underlying these events require further elucidation.

### EMT

EMT refers to the cellular phenotype change, in which epithelial cells acquire mesenchymal properties and attain motility. The EMT phenotype promotes attachment to the ECM. It enhances the migratory potential of an individual tumor cell by reorganizing actin fibers, which confers mesenchymal traits that are extremely important for tumor cells to efficiently intravasate into basement membranes and lymphatic vessels. In fact, infiltrating tumor cells undergo “partial” or “hybrid” EMT, leading to a mixed phenotype with both epithelial and mesenchymal traits, which favor collective over single-cell infiltration (52). In the tumor context, EMT is induced by various signals, such as transforming growth factor β (TGF-β), WNT/β-catenin, AKT signaling, inflammation, and hypoxia, which activate EMT-associated transcription factors including Snail, Twist, and Zeb family members (49, 53–59). These transcription factors trigger EMT, converge at the promotion of E-cadherin repressors, and mediate phenotypic changes by downregulating epithelial traits while inducing mesenchymal characteristics (60). Ultimately, the expressed cytoskeletal-remodeling and ECM-degrading proteases enable tumor cells to invade the surrounding tissue (61, 62).

Numerous EMT-inducing factors in the TME are associated with an increased risk of metastasis and a worse prognosis. For instance, the elevated expression of fatty acid-binding protein 5 (FABP5), nucleolar and spindle associated protein 1 (NUSAP1), and the oncoprotein cancerous inhibitor of protein phosphatase 2 (CIP2A) in the tumor tissue of CC patients is significantly associated with lymph node metastasis. Furthermore, the effects of knockdown or overexpression of these factors in different CC cell lines demonstrate that these molecules promote EMT by activating NF-κB signaling, Wnt/β-catenin signaling, and MEK/ERK signaling pathway, respectively (63–65). The long noncoding RNAs taurine-upregulated gene 1 (TUG1) and steroid receptor RNA activator (SRA), microRNA-21 and microRNA-663b modulate the EMT process and ultimately promote local and distant metastasis (66–69). Additionally, overexpressed PD-L1 binds directly to integrin β4 (ITGB4) and activates the AKT/GSK3β signaling pathway, consequently inducing the expression of Snail (56). PTPRM promotes EMT *via* the Src-AKT signaling pathway (49). Indeed, cancer cells are not the only cells that can induce EMT; CAFs also induce EMT by overexpressing TGF-β1 and stromal cell-derived factor 1 (SDF-1), further promoting the growth, invasion, and migration of CC (70). Taken together, these findings indicate that significant EMT in the TME is involved in lymphatic metastasis by promoting tumor adhesion and intravasation into the lymphatic vessel endothelium.

### ECM remodeling

Tumor metastasis requires the breakdown of the basement membrane and remodeling of the ECM. The proteolytic cleavage and degradation of ECM proteins are coordinated by a number of enzymes, including serine proteases, matrix metalloproteinases (MMPs) (71–73), and cysteine cathepsins (74). Alterations in these ECM molecules can be observed in the TME during tumor metastasis. For instance, elevated expression of MMPs has been reported in invasive CC tissues compared with normal cervical tissues, especially MMP-3 and MMP-9 (75). MMPs play a role in the degradation of the ECM through direct action. As one of the cysteine proteases, cathepsin L not only reduces cell adhesion through cleavage of E-cadherin but also degrades the ECM to support tumor migration and invasion. Moreover, increased cathepsin L expression in CC tissues correlates with poorer 5-year overall cumulative survival in CC patients (74).

These proteolytic enzymes can be secreted by tumor cells. However, the majority are secreted by resident stromal cells, such as cancer-associated myofibroblasts (myCAFs) (76). MMP-1 and MMP-2 secreted by CAFs play vital roles in initiating the invasion of malignant tumors and make tumor intravasation easier by clearing ECM proteins that block access to the endothelium. CAFs also promote the migration of tumor cells that express laminin receptors by facilitating the synthesis of laminin-1, which has been proven to be an efficient chemoattractant (71). Additionally, POSTN and lysyl oxidase-like 1 (LOXL1) overexpressed by CAFs degrade the basement membrane and stromal ECM and initiate the invasion of malignant tumors. Researchers have found that POSTN-expressing CAFs can impair lymphatic endothelial barriers by activating the integrin-FAK/Src/VE-cadherin signaling pathway in lymphatic endothelial cells. The impaired lymphatic endothelial barrier facilitates tumor cell intravasation and transendothelial migration, consequently promoting lymphatic metastasis in CC. Consistently, high stromal POSTN expression is closely associated with lymph node metastasis and low overall survival in CC patients (39). Overall, the abnormal metabolism of ECM components results in disturbed ECM homeostasis in the TME, which promotes stromal reconstruction and influences the infiltration of inflammatory components. Then, active ECM remodeling in the TME offers the necessary prerequisites for local invasion and distant metastasis by clearing the physiological barrier. In summary, the above five characteristics of the TME may promote lymphatic metastasis of CC in different but integrative ways.

## Diverse cells and intercellular interactions in the TME

**Figure 2:**
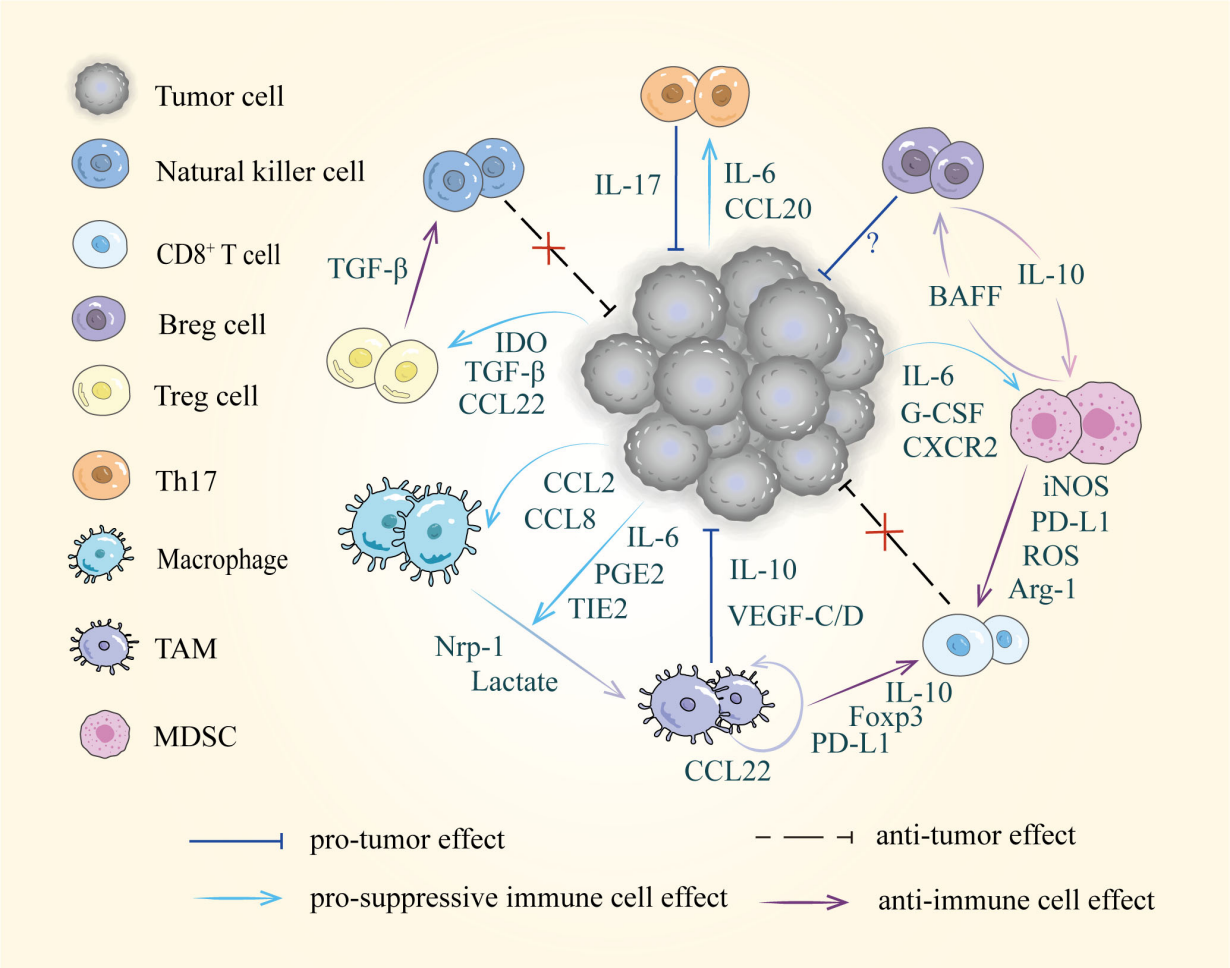
Diverse cells and intercellular interactions in the TME. Tumor cells recruit suppressive immune cells *via* released factors including cytokines, chemokines, and metabolic products, such as IL-6, CCL22 and IDO etc. Furthermore, these pro-tumor cells disrupt immune surveillance by suppressing the activity of natural killer cells and CD8^+^ T cells.

With further investigation of tumor metastasis, the crucial role of the TME has gradually been revealed. Primary tumor-suppressive immune cells can be converted into tumor-promoting cells in the TME. Immune cells display significant diversity and plasticity in response to stimulatory or suppressive cytokines (77). These immune cells, including T cells, B cells, TAMs, and MDSCs that undergo phenotypic changes can exert immunosuppressive effects through their surface receptors and released cytokines, creating a tolerogenic microenvironment in the tumor-draining lymph nodes that allows tumors to grow and metastasize (12) (**Figure 2**).

### T cells

Cellular immunity mediated by T lymphocytes is the cornerstone of antitumor immunity. However, tumor progression and metastasis are usually accompanied by a remarkably compromised antitumor response. The immunosuppressive TME driven by the close interactions between cancer cells and T lymphocytes contributes to tumor progression and forms a barrier that triggers resistance to immunotherapy. As mentioned before, tumor cells not only block the normal function of effector immune cells but also recruit suppressive lymphocytes. The metastatic lymph nodes in CC patients are characterized by the accumulation of PD-L1^+^ CD14^+^ antigen-presenting cells (APCs), FOXP3^+^ Treg cells, and PD-L1^+^ myeloid cells (37, 78). Furthermore, both CC cells and APCs at the invasive front express indoleamine 2,3-dioxygenase (IDO), which can recruit FOXP3^+^ Treg cells (79, 80). Tumors also promote Treg cell expansion by secreting exosomes that carry abundant signaling molecules, such as TGF-β, cyclic GMP-AMP synthase, and 2’-3’-cGAMP. Then, these exosomes activate the T cell-intrinsic stimulator of interferon genes (STING) signaling, resulting in immune suppression and poor outcomes in CC patients (81). In addition, the level of Th17 cells gradually increases with tumor progression, which is associated with an advanced clinical stage and lymph node metastases (82, 83). Mechanistically, CC cells indirectly support Th17 cell recruitment by instructing fibroblasts to produce high levels of CCL20 *via* the CCAAT/enhancer-binding protein β pathway (31). Besides, tumor-derived interleukin 6 (IL-6) supports IL23-mediated Th17 cell expansion (84).

In addition to aiding the immune evasion of tumor cells, immunosuppressive lymphocytes promote tumor proliferation and aggression. For instance, Th17 cells can promote angiogenesis, tumor proliferation, and invasion by secreting IL-17 and activating the NF-κB signaling pathway (85). Treg cells obtained from CC patients were shown to suppress the immune function of natural killer cells through the TGF-β pathway *in vitro* (86). Collectively, the close interactions between tumors and T cells result in a suppressive or even protumor immune state.

### B cells

Compared with T cells, the role of B cells in antitumor immunity remains poorly understood. B cells are principally known for their involvement in humoral immune responses. However, B cells from primary tumors are functionally different from those in the lymph node. In CC tumor-bearing mice, the percentage of B cells in the draining lymph nodes progressively increased with tumor growth. But there were few B cells in the primary tumor, and most of the tumor-infiltrating lymphocytes were T cells. These accumulated B cells were characterized by the overexpression of PD-L1 and the downregulation of MHC II molecules and participated in establishing an immunosuppressive TME. After being shaped by the microenvironment of draining lymph nodes, B cells promoted tumor growth in an IL-10-independent manner (87). More recently, studies on CC patients showed that elevated CD19^+^ CD5^+^ CD1d^+^ Breg cell levels were associated with lymph node metastasis and might be involved in the immunoregulation and inhibition of CD8^+^ T cells. *In vitro*, after coculture with Breg cells, the amounts of perforin and granzyme B secreted by CD8^+^ T cells were significantly decreased (88). Conversely, other studies have revealed an antitumorigenic role for B cells in HPV-related cervical squamous cell carcinomas and a remarkably beneficial impact on patient outcomes (89). Overall, B cells in the TME may possess dual functions. Specifically, B cells within the lymph nodes have potential immunoregulatory properties, and the ones in the primary CC tissue may play a key role in tumor control. However, due to the small amounts of B cells in the primary tumor, they exert limited antitumor effects.

### TAMs

In the TME, tumors actively recruit macrophages in various ways. For instance, CCL2 expressed by CC cells is associated with TAMs at the tumor site (90). Similarly, CCL8 derived from cancer cells attracts macrophages by binding to the receptor C-C chemokine receptor 2 (CCR2) on macrophages, which is related to the progression of CC. Notably, in the process of TAM migration, the hypoxia-induced transcription factor ZEB1 activates the transcription of CCL8, which is more important than CCL2 and macrophage colony-stimulating factor (M-CSF) (91). Macrophages possess a certain degree of plasticity that is regulated by microenvironmental stimulation. CC cells induce the polarization of macrophages into M2-like TAMs, which produce smaller amounts of proinflammatory cytokines, such as tumor necrosis factor α (TNF-α), IL-1β, IL-6, and nitrogen oxide (NO), but greater amounts of anti-inflammatory cytokines, such as IL-10 (92, 93). Tumor cells induce M2 polarization in macrophages by secreting various cytokines and growth factors. A blocking study revealed that M2 differentiation was caused by tumor-produced prostaglandin E2 (PGE2) and IL-6. However, TGF-β, IL-10, VEGF, and M-CSF did not play a role (94). Recently, research has confirmed that the nuclear factor of activated T cell 1 (NFATc1), a member of the Wnt family, is highly expressed in the CC microenvironment (95). It promotes M2 polarization of TAMs by regulating IL-10 secretion mediated by the c-myc/PKM2 pathway, thereby promoting tumor growth, invasion, and metastasis (96). Moreover, TAMs can promote their polarization toward M2a macrophages by secreting CCL22 in CC (97). In addition to the abovementioned mechanisms, tumor cells modulate metabolites to induce TAMs. For example, neuropilin-1 (Nrp-1) accumulation in the hypoxic TME educates recruited macrophages to polarize into the M2-phenotype (25). Lactate secreted by CC cells mediates crosstalk between tumor cells and macrophages, which promotes the secretion of IL-1, IL-10, and IL-6, and upregulates the expression of hypoxia-induced factor-1, further promoting a suppressive phenotype (98). Moreover, researchers have observed a direct transfer of tyrosine kinase with immunoglobulin and epidermal growth factor homology domains 2 (TIE2) proteins from TIE2-high CC cells to monocytes and macrophages *via* exosomes, which promoted increased infiltration of M2-like TAMs (99). All these findings suggest that CC cells in the TME actively recruit normal macrophages and induce them to serve as immunosuppressive TAMs by secreting various cytokines and manipulating metabolites.

Under the influence of tumor-derived factors, the TME is dominated by M2-like TAMs (30). Furthermore, TAMs modulate the immune response to facilitate tumor growth and progression. M2-like macrophages from HPV16-associated tumor-bearing mice were shown to induce a regulatory phenotype in CD8^+^ T lymphocytes by expressing IL-10 and Foxp3 (30). TAMs also recruit Treg cells by secreting the chemokine CCL22, and high expression of CCL22 is associated with poor outcomes in CC patients (80). PD-L1^+^ TAMs, which have been identified in different histological subtypes of CC, exert immunosuppressive effects by binding to PD-1 expressed on T lymphocytes (100). Moreover, studies have demonstrated that increased levels of macrophages in the tumor stroma are significantly associated with peritumoral lymphangiogenesis and lymphatic metastasis in CC. TAMs induce lymphangiogenesis by increasing the production of VEGF-C/D and VEGF-A, which stimulate the division of preexisting lymphatic endothelial cells (26, 101). Recently, a novel metastasis-promoting lymphatic pattern was found in CC, wherein TAMs encapsulated lymphatic vessels to form an interconnected network. After the formation of this pattern, the lymphatic vessels acquired an advantageous metastatic capacity. TAMs adjacent to lymphatic vessels could secrete IL-10 within the hypoxic TME, which increased the expression of Sp1 in LECs. As a transcriptional regulator in LECs that contributes to CC metastasis, Sp1 promoted the transactivation of CCL1 to facilitate TAMs and tumor cell recruitment further, forming a positive feedback loop to strengthen the pattern (102). In short, accumulated TAMs not only cooperate with CC cells to develop an immunosuppressive TME but also participate in lymphatic metastasis by inducing lymphangiogenesis.

### MDSCs

MDSCs are a group of heterogeneous immature myeloid cells with blocked maturation in cancer. They are one of the main driving forces of immunosuppressive TME (16). According to differences in phenotype and morphology, these cells can be divided into two subsets: granulocytic MDSCs (G-MDSCs), which resemble neutrophils, and monocytic MDSCs (Mo-MDSCs), which are similar to monocytes (103). An increased number of MDSCs in CC patients was first demonstrated in 2014 (104). Since then, a growing number of reports have indicated that the number of MDSCs, especially G-MDSCs, is significantly increased in the peripheral blood (35, 105–107), lymph nodes (36, 78), and tumor tissues of CC patients (108–110), which is associated with visceral or lymph node metastases (35, 78). In mouse models, MDSC depletion by splenectomy or anti-Gr-1-neutralizing antibody administration not only inhibited visceral organ metastasis and premetastatic niche formation but also prolonged the survival of CC-bearing mice (35, 104, 108, 109). In the TME of CC, tumor cells secrete various molecules to recruit MDSCs from immature bone marrow cells, including granulocyte colony-stimulating factor (G-CSF) (35, 36, 107, 108), IL-6 (111), and highly expressed C-X-C chemokine receptor 2 (CXCR2) chemokines, such as CXCL1, CXCL2, CXCL3, CXCL5, and CXCL8 (110).

MDSCs can inhibit T-cell proliferation and cytotoxic function by suppressing IL-2 and interferon γ (IFN-γ) production in CD4^+^ T cells and IFN-γ production in CD8^+^ T cells (105, 106, 109). Activated MDSCs secrete immunosuppressive factors, such as inducible nitric oxide synthase (iNOS), reactive oxygen species (ROS), IDO, and arginase I (Arg-1) to inhibit CD8^+^ T cells (28, 106, 112). It remains unclear by what mechanism and to what degree these factors derived from MDSCs lead to T-cell suppression. However, it is known that these factors lead to abnormal amino acid metabolism and the production of NO, resulting in diminished T-cell proliferation and impaired antigen presentation and recognition by CD8^+^ T cells (113). Using an HPV-mediated CC mouse model, researchers demonstrated that MDSCs mediated immunosuppressive activity *via* IL-6/JAK/STAT3 signaling. STAT3 activation mediated by the proinflammatory cytokine IL-6 might be responsible for the expansion of MDSCs, which then accelerated tumor growth (112). Moreover, MDSC-derived IL-6 was reported to be partly involved in stimulating tumor cell proliferation (111, 114). Additionally, MDSCs interact with B lymphocytes in the TME of CC *via* the B cell activating factor (BAFF) expressed on the surface of MDSCs. By acting on the BAFF receptor expressed by B cells, MDSCs induce B cells to differentiate into Breg cells. Furthermore, IL-10 secreted by Breg cells can promote STAT3 phosphorylation and activate MDSCs, thus establishing a positive feedback loop. The constant differentiation of Breg cells and activation of MDSCs induce an immunosuppressive state and enable tumor immune escape in CC patients (28).

Studies of mouse models have revealed that MDSCs also contribute to premetastatic niche formation (36), tumor angiogenesis (107), and the enhancement of the stem-like properties of cancer cells (108), all of which facilitate tumor metastasis (35). For instance, MDSCs have been reported to enhance the stemness of CC cells by producing PGE2. MDSC depletion inhibited the induction of cancer stem-like cells and enhanced the efficacy of chemotherapy in experimental models of CC (108). MDSCs also stimulate tumor angiogenesis by secreting Bv8, a potent proangiogenic factor (104, 107). In summary, MDSCs utilize several mechanisms to enhance the proliferation and metastasis of CC. After being recruited to the TME, they exert powerful immunosuppressive effects mainly by suppressing T-cell function.

## Therapeutic strategies for targeting the TME of cervical cancer

Over the past several years, standard treatments for patients with advanced CC have included radiotherapy and chemotherapy, but their prognosis has been disappointing (115). Therefore, researchers focus on exploring new treatment options, among which immunotherapy is considered promising. The importance of immunotherapy has been highlighted in recent years as further insights into the interactions between tumors and immune cells in the TME have been reported. To date, three immunotherapeutic drugs have been approved by the Food and Drug Administration (FDA) for treating CC: pembrolizumab, tisotumab vedotin, and nivolumab. Furthermore, various immunotherapeutic strategies are being developed to reactivate the antitumor immune responses or remodel the immunosuppressive microenvironment. Therefore, we briefly summarize the progress in therapeutic strategies targeting the TME of CC.

### Targeting immune checkpoint molecules

In particular, the upregulation of immune checkpoint molecules in tumor tissues, such as PD-L1 and PD-1, promotes immune escape by downregulating T-cell function. Anti-PD-1 therapies have been demonstrated to enhance the disease-free progression and survival rates of CC patients (116, 117). Although the FDA has approved several anti-PD-L1 antibodies for tumor immunotherapy, none have yet been approved for treating CC. A clinical trial performed with patients with recurrent or metastatic CC indicated that the anti-PD-L1 monoclonal antibody (mAb) socazolimab has durable safety and efficacy (118). Several other trials have demonstrated the therapeutic benefit of immune checkpoint inhibitors (ICIs) in CC, as reviewed in (119, 120). However, ICI monotherapy in CC yields limited durable responses. A few relevant clinical trials are ongoing to find more effective therapies or combination regimens. Recently, a bifunctional agent bintrafusp α was developed to target PD-L1 and TGF-β simultaneously, and one CC patient demonstrated a durable complete response in this phase I clinical trial (121). Rather than solely targeting immunosuppression in the TME or enhancing effector immune cells, combining multiple strategies may achieve better outcomes. The preliminary data of a phase I clinical trial combining an anti-PD-L1 mAb (durvalumab) with anti-cytotoxic T-lymphocyte associated protein 4 (CTLA4) (tremelimumab) and metronomic chemotherapy reported that one CC patient achieved a complete response and four patients achieved stable disease, and a phase II trial is currently ongoing (122). Except for the PD-1/PD-L1 axis and CTLA4, other therapeutic antibodies aiming to activate T cells and eliminate the inhibition of T-cell activation are being tested. For example, single-agent human agonistic mAbs specific for OX40 and 4-1BB, such as ivuxolimab or utomilumab, have demonstrated safety and preliminary antitumor activity in CC patients. OX40 and 4-1BB belong to the tumor necrosis factor receptor superfamily and play important roles in T-cell activation, proliferation, and survival. Costimulating these molecules induces clonal expansion of CD4^+^ and CD8^+^ T cells, as well as increased cytotoxicity of T cells (123). Nevertheless, no such treatment has yet been approved for application in CC.

### Targeting suppressive immune cells

Due to the existence of suppressive immune cells in the TME, compromised efficacy or resistance to ICI therapy may occur. Therefore, therapeutic approaches targeting suppressive immune cells, such as MDSCs and TAMs, are being tested for their capacity to improve sensitivity to ICIs. In fact, the antitumor effects of anti-PD-1 therapy are enhanced by inhibiting the main chemokine receptor CXCR2 that recruits MDSCs in human pancreatic cancer (124). Research on CC also indicates that treatment with a CXCR2 antagonist weakens the proliferation and migration of CC cells (125). Additionally, the approaches targeting the CSF-1/CSF-1R axis of TAMs are being tested in mouse models. CSF-1R inhibition attenuates the turnover rate of TAMs while increasing the number of CD8^+^ T cells that infiltrate tumor tissue (126). Although these are results from only cellular and animal experiments, they bring forward new ideas for the treatment of CC. As mentioned before, various metabolites derived from suppressive immune cells, such as Arg-1 and IDO, contribute to the impaired function of effector T cells. Thus. limiting certain metabolites holds promise. Recently, the arginase inhibitor INCB001158 was tested in a clinical trial for metastatic solid tumors treatment (NCT02903914). Another immunosuppressive metabolite, IDO, within the TME is also speculated to be a worthy target for intervention. Treating IL-6 knockout mice with IDO inhibitors was shown to inhibit IDO expression. Furthermore, combination therapy with a therapeutic vaccine resulted in decreased intratumoral polymorphonuclear MDSCs and Treg cells, supporting IL-6 and IDO as immune-metabolic adjuvants for immunotherapies against CC (127). Considering this preclinical research data, it appears that targeting the suppressive immune cells and metabolites within the TME can be used in combination therapies to boost tumor-specific immunity, further improving the therapeutic antitumor effect.

### Anti-lymphangiogenesis and anti-inflammatory therapy

Currently, chemotherapy in combination with bevacizumab, an antiangiogenic molecule, is the first-line therapy for recurrent or metastatic CC. In contrast, the search for a therapeutic agent that explicitly inhibits lymphangiogenesis has yet to achieve a breakthrough. As lymphangiogenesis and lymphatic remodeling are functionally important in tumor progression, they represent potential targets for treating metastatic CC. The VEGF-C/VEGFR-3 signaling axis is the most prominent and specific pathway that induces tumor lymphangiogenesis (14, 26, 38, 41). Blocking VEGF-C/VEGFR-3 has been shown to reduce tumor lymphangiogenesis and metastasis in experimental models (128). However, due to the scarcity of relevant studies in CC, the exact effects of targeting lymphangiogenesis are still unclear, and more research is needed.

Since chronic inflammation is another essential contributor to tumorigenesis and metastasis, anti-inflammatory drugs may be a promising tool. The combined effect of nonsteroidal anti-inflammatory drugs with chemotherapy and radiotherapy has increased sensitivity in patients with locally advanced CC. Therapeutic targeting of the COX/PGE2 axis in CC has been reviewed (129). In addition, several anti-inflammatory drug candidates can potentially be therapeutic agents for CC, but further experimental validations are needed (130). Currently, lymphangiogenesis and inflammation-inhibiting therapies are only used as supplements to chemoradiotherapy and immunotherapy. The interactions of various cells in the TME are so complicated that the effect of any single therapy is limited. An increasing number of clinical trials are focusing on novel combination therapies, which are expected to significantly improve the outcome and prognosis of patients with recurrent or metastatic CC.

### Nanomaterial-based photodynamic therapy

Nanomaterial-based photodynamic therapy (PDT) has emerged as a novel noninvasive and highly selective treatment for cancer. Photosensitizers enriched in tumor sites are activated by light to sensitize endogenous molecular oxygen and generate cytotoxic ROS, which induces apoptosis or necrosis in tumor cells. However, PDT efficiency is significantly limited because ROS-mediated therapy heavily depends on O_2_, while TME is hypoxic. A novel nanoagent, copper ferrite nanospheres (CFNs), has been reported to exhibit enhanced ROS production. CFNs reduce the hypoxia and antioxidant activity of tumors by catalyzing the high content of H_2_O_2_ in the TME to produce O_2_ and consume glutathione, improving the effects of PDT. Moreover, CFNs have shown considerable anticancer effects *in vitro* and *in vivo*. After treating xenograft CC cell (U14) tumor models with CFNs, tumor tissues were damaged, and tumor growth was apparently inhibited (131). Recently, an oxygen-independent supramolecular photodynamic agent was developed to oxidize water to generate highly cytotoxic hydroxyl radicals directly. This agent exhibited superb photocytotoxicity even under severely hypoxic environments and excellent antitumor efficacy in HeLa tumor-bearing mouse models (132).

In addition to nanoparticles that kill cancer cells *via* ROS, other nanoparticles with different functions have been developed. A synthetic therapeutic antibody based on molecularly imprinted nanoparticles (MIP-NPs) has been designed to abrogate cell-cell adhesion. The abolishment of cell adhesion by these antibodies inhibits primary tumor growth and metastatic progression, although the mechanism is not completely understood. Remarkably, when applied to CC (HeLa) cells, MIP-NPs disrupted preformed tumor spheroids and inhibited cancer cell invasion *in vitro*. Moreover, the dense ECM in the TME makes it difficult for drugs to penetrate tumors. MIPs, an antibody against cadherins, can help to loosen cells to allow more effective drug penetration (133). Because of the high tumor interstitial pressure (TIP), delivering nanodrugs to tumors can be challenging. Considering this situation, researchers fabricated the photocatalytic drug AWS@M, which effectively reduced TIP levels, to enhance intratumoral drug delivery and inhibit tumor growth in U14 tumor-bearing mice (134). Nanomaterials are a prospective agent in tumor therapy, with new nanoagents constantly being developed to achieve excellent biocompatibility and higher selectivity. The combination of multiple approaches, including immunotherapy, targeted therapy, and PDT, is expected to improve tumor-targeting efficiency and capability and bring hope to cancer patients.

## Conclusion and future perspectives

Over the past decade, the field has reached a consensus on the critical roles of the TME in tumor progression and metastasis. Generally, CC cells actively recruit immunosuppressive cells, such as Th17 cells, Treg cells, TAMs, and MDSCs, into the TME. These cells hamper immunosurveillance and weaken therapeutic effects by participating in multiple pathological processes. Recent studies have shown that tumor-derived exosomes mediate nonrandom dissemination patterns by biasing metastasis to different target organs due to their affinity for specific recipient cells. However, evidence in CC is still scarce, and it is difficult to determine how CC cells preferentially metastasize to the lymph nodes. Many significant issues regarding premetastatic niche formation, dynamics, and lymphangiogenesis in CC remain unanswered. Regarding the mechanism research of tumor-immune cell interactions involved in the lymphatic metastasis of CC, some limitations must be addressed. First, most preclinical studies have not specifically tested metastatic CC or with lymphatic metastasis as an end-point. Of note, most CC-related tumor biology studies have explored only the effects of certain types of immune cells in tumor progression. An in-depth exploration of the underlying mechanisms is still needed. Second, the degree to which lymphangiogenesis directly contributes to lymphatic metastasis and the mechanism through which this occurs needs to be defined. Third, due to the significant heterogeneity among tumor cells and immune cells in the TME, as well as the multidirectional and pleiotropic effects of cytokines and growth factors, it is difficult to determine the roles played by individual immune cell subsets in the overall tumor progression process.

In summary, a part of the complex interactions between tumors and various immune cells has been gradually unveiled, but a large part remains unknown, and more research is needed. A better understanding of the mechanisms driving tumor lymphatic metastasis will contribute to more precise targeting of vital pathways or molecules, enabling better clinical outcomes for CC patients.

## Author contributions

MW conceived the concept and made the final approval of manuscript. YL investigated literatures and wrote the manuscript. XG, YH, and BW revised the manuscript. XZ, QZ, LX, and YC designed the figures. All authors listed made substantial, direct intellectual contributions to the work, and approved it for publication. All authors contributed to the article and approved the submitted version.
